# Depression as an initial feature of Systemic Lupus Erythematosus? A case report

**Published:** 2010-05-25

**Authors:** G Marian, EA Nica, BE Ionescu, DG Carlogea

**Affiliations:** * UMF Carol Davila, ‘Prof. Dr. Al. Obregia’ Clinical Hospital, BucharestRomania; ** ‘Prof. Dr. Al. Obregia’ Clinical Hospital, BucharestRomania; *** ‘Ana Aslan ’ INGG, BucharestRomania

**Keywords:** chronic illness, Systemic Lupus Erythematosus, depression

## Abstract

Many patients with chronic illnesses suffer from clinical depression (with percentages reported by clinical studies ranging from 15 to 60); even depression is more common in people with chronic medical illnesses, (e.g. Systemic Lupus Erythematosus) than in the general population. However, not every patient with a chronic illness suffers from depression. It is well known that some people think the persons who suffer from a chronic illness have a reason to be depressed, so, there is no need to go to a psychiatrist for treatment. The fact is that not only the medical condition can be the cause for depression and, not always, the treatment for a medical illness can supply the treatment for depression. The condition of clinical depression, associated with a chronic illness, needs to be early diagnosed and early treated because it can worsen the medical state. Systemic Lupus Erythematosus (SLE) is a chronic, autoimmune disease, which affects many organs and systems. The link between lupus and depression is controversial but it is known that negative life events, lupus activity and the treatment for disease may be capable of contributing to clinical depression. We present the case of a young woman who was diagnosed with Systemic Lupus Erythematosus and who initially presented in the psychiatric department for depressive symptoms. The case raised problems in terms of diagnosis, treatment, etiology and prognosis.

## Introduction

Systemic lupus erythematosus is a multi-systemic disease of unknown etiology [1]. SLE is an autoimmune disease; the condition may vary widely in severity, from a simple skin rash, headaches to chorea and permanent paralysis. Neuro–psychiatric symptoms are frequent in SLE with a range between 13% to 75%.  The psychiatric symptoms may vary from mild personality disorders to severe psychotic behavior. Shearn and Pirofsky found that endogenous depression is the most frequent mental change in lupus (about 50% of the patients develop depression) [2]. The management of depression in lupus rests on a combination of treating the underlying lupus itself as well as adding antidepressant therapy.

## Case presentation

CD, 24 years old, university student, female

The patient smoked but evidently did not abuse of drugs or alcohol and was not taking any drugs before admission. The patient had no history of psychiatric illnesses; there was no known family history of psychiatric or autoimmune diseases.

One month prior to the admission, she became sad, cried easily, had headaches, lost interest in the daily activities, presented heart palpitations and disturbance in sleep and appetite. The symptoms were worse day by day and she decided to call for a psychiatric consult.

### Psychiatric consult

Patient was properly dressed, according to time and place. She was in a depressant mood, she had anhedonia, psychomotor retardation, thoughts of helplessness, hopelessness, low self–esteem, insomnia, fatigue. The patient said that she was unable to think or to act, but memory functions were not impaired. No auditory or visual hallucinations were detected

### Physical examination

Photosensitive butterfly rash on the face (the symptoms of malar rash and photosensitivity have been noted for about 5 months, by herself) and vasculitis lesions over her hands. She had alopecia and Raynaud's phenomenon over both hands. Eye, ear and nose– in normal ranges. No lymphadenopathy was found. Oral ulceration of 1 cm diameter over the soft palate. Pain and restriction of the movement in her joints were found.

Heart and lungs–no significant abnormality

No palpable spleen and liver.

### Laboratory results

- WBC count: 8,150//dL;
- Hb 10,2 gm/dL;
- ESR– 58 mm/hr;
- Platelet– 255,000/ mm^3^;
- EEG was normal;
- CT was normal;

Suspecting an autoimmune disease, we asked for supplementary investigation:

- rheumatoid factor negative
- C3: 72,4 mg/dL (90–180mg/dL)
- C4:32 mg/dL (10–40mg/dL)
- T3, T4, TSH in normal ranges
- L.E. cell was positive
- Anti nuclear antibodies (ANA) negative

Taking into account all the dates, we started the treatment with Diazepam 10mg/day. Subsequently, she started the treatment with Prednisolone 60 mg/day and was sent to a medical clinic in order to have her evolution tracked. In a few days from the beginning of the treatment with Prednisolone, the patient developed other psychiatric symptoms such as agitation; logorrhea, suicidal ideation, confusion and illusions. The diagnosis was steroid induced psychosis and 2 mg of risperidonum was given. The symptoms disappeared completely in approximately 2 months, in conditions of prednisone dosage reduction. The treatment with Risperidonum was interrupted after 2 months. After this episode, no psychotic symptoms were present. 

After 3 months of follow–up the depressive symptoms persisted, and an antidepressant treatment was started (Duloxetine 60mg/day). After another 6 months with prednisone 5 mg/day and Duloxetine 60mg/day, the dose of Duloxetine was decreased at 30 mg/day and after 2 months of stable mood and no significant symptoms of depression, it was eliminated. 

## Discussion

1. The case made the diagnostic criteria of the American Rheumatism Association [3] for SLE (alopecia, facial erythema, positive L.E. cells, oral ulceration, vasculitis and Raynaud's phenomenon). What was unusual in this case is the fact that anti nuclear antibodies (ANA) were negative (a small group of patients develop clinical features remarkably similar to those of SLE, but never appear to develop ANA [4]). In our case, a test made after the initiation of the treatment with Prednisone showed ANA (+).
2. Steroid psychosis often occurs from a few days to 2 weeks after the administration of this agent. Patients with steroid–induced psychosis often show mood disorders including logorrhea, distraction, confusion and hallucination. In our case, two factors of the ones which are considered to be major risk factors for steroid psychosis, namely being female and being administered Prednisolone 60 mg/day (more than 40 mg/day), were present. [5,6]
  The treatment of steroid psychosis involves dosage reduction or discontinuation of Prednisolone, but this usually cannot be done due to the severity of the underlying disease that requires Prednisolone. In such cases, we have to treat the patient with antipsychotic drugs. Tricyclic and tetracyclic antidepressants have been reported to induce exacerbation of agitation [6–8]. It is difficult to distinguish psychotic episodes caused by steroids from those caused by central nervous system disorders in collagen, but symptoms' disappearance when the dose of Prednisone was decreased, made things clear about the symptoms developed shortly after the initiation of the treatment.
3. The depressive episode is hard to be interpreted, because it was the reason that made the patient come to the doctor, however, it was not the only disorder found. The etiology of psychiatric manifestation of SLE is still unknown. We cannot say if it is a simple coincidence or the psychiatric symptoms can be explained as a part of the disease spectrum of SLE. The depression in SLE is frequent and it can be caused by the lupus itself, by various medications used to treat lupus and by other factors unrelated to lupus. Psychiatric disorders may merely be reactive psychological disturbances due to the stress related to the impact of having a chronic illness [9,10]. Supportive psychotherapy has been successful in helping many patients with SLE adjust to the limitation imposed by the disease [11].

## Conclusions

When a patient comes to a psychiatrist for some psychiatric symptoms we have to remember that a responsible physical examination must be done, because is easy to miss an organic disease. In our case, we can say that we are not talking about psychiatric symptoms as an initial feature of SLE, because the patient herself noted the malar rash, some months before she started having depressive symptoms. When we suspect a medical illness in our patients, we must collaborate with the other specialists in order to make an early diagnosis, which will lead to an early intervention. The treatment for depression in lupus consists of psychotropic medication, psychotherapy, and, most often a combination of both.
